# Human APOE4 increases microglia reactivity at Aβ plaques in a mouse model of Aβ deposition

**DOI:** 10.1186/1742-2094-11-111

**Published:** 2014-06-19

**Authors:** Gustavo A Rodriguez, Leon M Tai, Mary Jo LaDu, G William Rebeck

**Affiliations:** 1 Department of Neuroscience, Georgetown University Medical Center, 3970 Reservoir Road, NW Washington, DC 20057, USA; 2 Department of Anatomy and Cell Biology, University of Illinois at Chicago, Chicago, IL, USA

**Keywords:** Neuroinflammation, Microglia, Alzheimer’s disease, Aβ_1–42_, APOE4, MOAB2

## Abstract

**Background:**

Having the apolipoprotein E4 (*APOE*-ϵ4) allele is the strongest genetic risk factor for the development of Alzheimer’s disease (AD). Accumulation of amyloid beta (Aβ) in the brain is influenced by APOE genotype. Transgenic mice co-expressing five familial AD mutations (5xFAD) in the presence of human APOE alleles (ϵ2, ϵ3 or ϵ4) exhibit APOE genotype-specific differences in early Aβ accumulation, suggesting an interaction between APOE and AD pathology. Whether APOE genotype affects Aβ-plaque-associated neuroinflammation remains unclear. In the current study, we address the role of APOE genotype on Aβ-associated microglial reactivity in the EFAD transgenic mouse model.

**Methods:**

We analyzed Aβ-induced glial activation in the brains of 6-month-old EFAD transgenic mice (E2FAD, E3FAD and E4FAD). Region-specific morphological profiles of Aβ plaques in EFAD brain sections were compared using immunofluorescence staining. We then determined the degree of glial activation in sites of Aβ deposition while comparing levels of the inflammatory cytokine Interleukin-1β (IL-1β) by ELISA. Finally, we quantified parameters of Aβ-associated microglial reactivity using double-stained EFAD brain sections.

**Results:**

Characterization of Aβ plaques revealed there were larger and more intensely stained plaques in E4FAD mice relative to E2FAD and E3FAD mice. E4FAD mice also had a greater percentage of compact plaques in the subiculum than E3FAD mice. Reactive microglia and dystrophic astrocytes were prominent in EFAD brains, and primarily localized to two sites of significant Aβ deposition: the subiculum and deep layers of the cortex. Cortical levels of IL-1β were nearly twofold greater in E4FAD mice relative to E3FAD mice. To control for differences in levels of Aβ in the different EFAD mice, we analyzed the microglia within domains of specific Aβ deposits. Morphometric analyses revealed increased measures of microglial reactivity in E4FAD mice, including greater dystrophy, increased fluorescence intensity and a higher density of reactive cells surrounding cortical plaques, than in E3FAD mice.

**Conclusions:**

In addition to altering morphological profiles of Aβ deposition, APOE genotype influences Aβ-induced glial activation in the adult EFAD cortex. These data support a role for APOE in modulating Aβ-induced neuroinflammatory responses in AD progression, and support the use of EFAD mice as a suitable model for mechanistic studies of Aβ-associated neuroinflammation.

## Background

Alzheimer’s disease (AD) is a progressive age-related neurodegenerative disorder, which results in declarative memory deficits and an impaired ability to function in daily life [1,2]. It is the most common form of dementia in the elderly and currently affects over 5 million people in the United States alone. Pathologically, it is characterized by the presence of numerous amyloid plaques and neurofibrillary tangles in the brain [3,4], with particular pathological effects appearing in the medial temporal lobe and cortex. Extracellular plaques are formed by the aggregation and deposition of amyloid beta (Aβ) peptides. Aβ aggregates are associated with dystrophic astrocytes and microglia as well as pro-inflammatory molecules that may exacerbate AD pathology [5-7]. Indeed, the clinical symptoms of AD correlate closely with neuronal damage, stemming in part by Aβ-driven neuroinflammatory responses [8,9].

Apolipoprotein E (*APOE*) is a polymorphic gene in humans (*APOE-*ϵ2, *APOE-*ϵ3 *APOE-*ϵ4), coding for three common protein isoforms that differ at positions 112 and 158 [10]. Although primarily associated with lipid and cholesterol homeostasis in the central nervous system (CNS) and periphery, APOE exerts genotype-specific effects on Aβ aggregation, metabolism, and plaque load in both AD patients and mouse models of AD [11-19]. Levels of Aβ found in the AD brain are strongly affected by APOE genotype [20-22], contributing to its role as a major genetic risk factor for AD [23-25]. Interestingly, APOE genotype also differentially affects glial-mediated inflammatory responses in the brain [22,26-30], suggesting that APOE-associated AD risk may in part be driven by dysfunctional neuroinflammation in response to Aβ pathology. At present, the effects of APOE genotype on Aβ-associated glial activation are unclear.

The EFAD transgenic mouse model was recently developed to investigate APOE-genotype-specific effects on AD pathological changes in the brain [31,32]. In this model, 5xFAD mice expressing transgenic forms of the amyloid precursor protein (APP) and presenilin-1 (PS1) [33] were crossed to APOE knock-in mice expressing each of the three APOE isoforms [34]. The resulting EFAD mice (E2FAD, E3FAD and E4FAD) exhibit robust amyloid deposition in the brain, even at young ages [32]. Young (2 to 6 month old) E4FAD mice exhibit accelerated Aβ accumulation in the subiculum and frontal cortex, greater total levels of Aβ_1–42_ in the hippocampus, and selective increases in soluble Aβ42 and oligomeric Aβ (oAβ) compared to E2FAD and E3FAD mice. Thus, EFAD transgenic mice are a tractable model of APOE-influenced extracellular Aβ deposition in the brain, and may be an attractive model for investigating the synergistic effects of APOE genotype and plaque deposition on neuroinflammation. Whether APOE genotype differentially affects Aβ-associated neuroinflammation in the brains of EFAD mice has not been addressed.

Glial-mediated inflammatory responses to Aβ are an important mechanism in AD pathogenesis, yet little is known regarding the role of APOE genotype in this process. In the current study, we examined the influence of APOE genotype on Aβ plaques and plaque-associated glial activation in 6-month-old EFAD mice using immunohistochemistry. We report that APOE4 had an effect on Aβ plaques in the brain, and that E4FAD mice exhibited an increased density of reactive microglia within cortical plaque domains, independent of overall plaque burden. These data suggest that APOE genotype differentially affects extracellular Aβ-associated microglial activation in the brain.

## Material and methods

### Animals

EFAD mice were used for all experiments and have been described previously [32]. Briefly, EFAD mice co-express five FAD mutations (APP K670N/M671L + I716V + V717I and PS1 M1461L + L286V) on backgrounds of homozygous *APOE2*, *APOE3* or *APOE4* knock-in genotypes. All EFAD mice were on a C57BL/6 J genetic background and were maintained in a temperature- and humidity-controlled vivarium at the University of Illinois at Chicago, provided food and water *ad libitum*, and subjected to a standard 12-hr light/dark cycle. All experiments were conducted in accordance with National Institutes of Health guidelines for the care and use of laboratory animals, and all protocols were reviewed and approved by the institutional animal care and use committees at the University of Illinois at Chicago.

### Tissue harvesting

Brain tissue was harvested as previously described [32]. Briefly, 6-month-old EFAD mice (E2FAD, *n* = 4; E3FAD, *n* = 5; E4FAD, *n* = 5) were deeply anesthetized with sodium pentobarbital (50 mg/kg) and perfused transcardially with ice-cold 100 mM phosphate-buffered saline (PBS) pH 7.4. Brains were dissected at the midline, with the left hemi-brains fixed in 4% paraformaldehyde for 48 hr, rinsed and stored at 4°C in PBS plus 0.05% sodium azide until use. Right hemi-brains were dissected on ice to give cortex, hippocampus and cerebellum samples, then snap frozen in liquid nitrogen, and finally stored at -80°C until use.

### Biochemical analysis of IL-1β levels in the cortex

Serial extraction of soluble proteins was performed as previously described [32]. Briefly, the dissected cortices were homogenized in 15 volumes (w/v) of tris-buffered saline (TBS), centrifuged (100,000 *g*, 1 hr at 4°C) and the TBS-soluble extract was frozen in liquid nitrogen and stored at -80°C. IL-1β levels were measured by ELISA (Life Technologies, Carlsbad, CA) according to the manufacturer’s instructions, and normalized to total protein levels (Quick Start™ Bradford Assay, Bio-Rad, Hercules, CA).

### Tissue processing for immunohistochemical analysis

Sagittal brain sections (30 μm) were sliced in ice-cold PBS using a Leica VT1000S vibratome and stored in cryoprotectant at -20°C until the immunostaining procedures. Sections processed for staining and analysis correspond approximately to plates 111, 115 and 119 in the Paxinos and Franklin mouse brain atlas [35], following a 1:9 series of tissue staining. For both peroxidase and fluorescence staining procedures, E2FAD, E3FAD and E4FAD brain sections were processed in parallel.

### Immunoperoxidase staining

Cryoprotectant was washed from free-floating tissue sections with PBS before quenching endogenous peroxidases with 3% H_2_O_2_. Sections were then blocked with 3% BSA and 10% horse serum in PBS for 1 hr at room temperature, followed by overnight incubation at 4°C with either Iba1 (1:5,000) or GFAP (rabbit, 1:500 dilution of 0.4 μg/μL stock) (Invitrogen, Carlsbad, CA) antibodies in blocking buffer. Tissue sections were rinsed and incubated with a biotinylated secondary antibody (goat anti-rabbit, 1:1,000 dilution of 1.5 mg/mL stock) (Vector Labs, Burlingame, CA) for 1 hr at room temperature. Sections were then incubated with an avidin-biotin conjugate (VECTA STAIN Elite ABC Kit, Vector Labs) before being developed in PBS containing 0.04% 3,3'-diaminobenzidine (DAB) hydrochloride and 0.04% nickel ammonium sulfate. After staining was complete, sections were mounted on glass Superfrost Plus slides, allowed to air dry for 24 hr, then dehydrated and cleared with xylene before being covered by a coverslip.

### Immunofluorescence staining

Prior to immunofluorescence staining, free-floating tissue sections were washed in TBS pH 7.4, then subjected to heat-mediated antigen retrieval for 10 min in 30 mM sodium citrate buffer (pH 6.0) containing 0.05% Tween 20. Sections were then permeabilized with 0.25% Triton X-100 in TBS (TBST) and blocked with 5% BSA in TBST for 1 hr at room temperature. Tissue sections were subsequently incubated with an anti-Aβ antibody, MOAB2 (mouse IgG_2b_, 1:1,000 dilution of 0.5 mg/ml stock) [32,36], and either an anti-microglia antibody, Iba1 (rabbit IgG, 1:5,000 dilution of 0.5 μg/μL stock) (WAKO Pure-Chemical Industries, Osaka, Japan), or an anti-astrocyte antibody, GFAP (rabbit IgG, 1:1,000 of stock) (Millipore, Billerica, MA), diluted in TBST containing 2% BSA overnight at 4°C. Next, sections were washed in TBST, then incubated with Alexa fluorophore-conjugated secondary antibodies (Invitrogen) diluted 1:1,000 in TBST containing 2% BSA for 1 hr at room temperature. After subsequent washing, tissue sections were mounted onto glass Superfrost Plus slides (Fisher Scientific, Pittsburgh, PA) using Fluoromount-G (Southern Biotech, Birmingham, AL), a coverslip was added and the slides were stored in the dark at 4°C until imaging.

### Image analysis

Immunoperoxidase-stained EFAD brain tissue was analyzed under bright field microscopy using an AxioPhot upright microscope (Zeiss Microsystems, Inc., Oberkochen, Germany). Digital images were acquired using an AxioCam HRm camera connected to a Dell computer running AxioVision 4.8.2 software (Zeiss Microsystems, Inc.). Region-specific gliosis was observed and qualitatively assessed in the subiculum and deep layers of the cortex by an investigator blinded to APOE genotype. Dystrophic astrocytes were readily identified by their large somas, hypertrophic primary processes and intense GFAP-immunoreactivity relative to astrocytes outside the subiculum and deep layers of the cortex. Reactive microglia were characterized by swollen cell bodies and intense Iba1-immunoreactivity. In separate experiments, *z*-stacked images of double-stained immunofluorescent EFAD brain sections were acquired and processed into two-dimensional projection images using a Zeiss 510LSM confocal microscope and the LSM Image Browser (Zeiss Microsystems, Inc.), respectively. Image files were then coded and analyzed in the freely available open-source image-processing suite Fiji by an investigator blinded to APOE genotype [37].

Morphological features of Aβ plaques and gliosis in EFAD brains were analyzed within individual fluorescent channels in Fiji and saved via the ROI Manager. Aβ plaques were defined as MOAB2-positive areas of stained tissue consisting of a single massed body at least 10 μm in diameter. Individual plaques within the subiculum and deep layers of the cortex were selected for analysis by an investigator blinded to APOE genotype. Plaques were traced to obtain the plaque area (μm^2^), fluorescence intensity (AU) and plaque type. Sampled plaques were classified into three major categories based on a previous report [32]: (a) diffuse, having no center and weak MOAB2 staining with a wispy morphology; (b) dense core, having an obvious center that stains brightly with MOAB2, and having weakly stained fibrils surrounding the core and (c) compact, having a very brightly stained core with no obvious MOAB2 stained fibrils, and generally smaller than other plaques (approximately 10 to 20 μm in diameter). Investigators sampled as many plaques as possible in an image window for analysis, resulting in over 200 plaques traced per APOE genotype. To obtain morphological measures of Aβ-associated microgliosis, 75-μm-diameter rings were centered over plaques to define plaque domains and then superimposed on images of Iba1-stained microglia. Plaques with little to no overlapping plaque domains were randomly selected under the same blinded conditions as plaque analysis, as was image analysis of microgliosis. Total Aβ-associated microglial density was calculated within each plaque domain, with a distinct cell soma required for cell counts. Iba1 fluorescence intensities were also calculated within each plaque domain. For each fluorescence channel, three measures of background fluorescence were collected and the average subtracted from the total fluorescence of individual plaques and plaque domains in each image.

Additionally, the extent of Aβ-associated microgliosis in EFAD sections were analyzed by thresholding the images and measuring the total area (μm^2^) occupied by Iba1-stained microglial cell bodies and processes within individual plaque domains. Minimum threshold values for 8-bit single-channel images of Iba1 staining were adjusted interactively (range 35 to 45) under blinded conditions in Fiji using an over/under display mode (blue is background). The aggregate Iba1 immunoreactivity above threshold was then measured within the superimposed plaque domains and saved as a percentage of total plaque domain area.

### Statistical analysis

All statistical analyses were performed using GraphPad Prism 5 unless noted otherwise. Differences between means were assessed by one-way analysis of variance (ANOVA), or two-way ANOVA followed by Tukey’s honest significant difference and Bonferroni *post hoc* tests, where appropriate. Descriptive statistics are expressed as mean ± standard error of the mean, with significance set at *P* < 0.05.

## Results

### APOE genotype affects Aβ plaque morphology in the EFAD subiculum and deep layers of the cortex

5xFAD-Tg mice exhibit a region-specific accumulation of Aβ in the brain as early as two months of age, with significant levels of thioflavin-S-stained plaques appearing in the hippocampus and cortex at four months [38]. Human APOE delays somewhat the formation of amyloid plaques in these mice and decreases total levels of Aβ_1–42_, and thus these mice are a tractable model for studying the effects of APOE genotype on Aβ deposition and Aβ-associated neuroinflammation [32]. To investigate this area, we performed experiments by staining for Aβ and microglia on tissue sections from 6-month-old EFAD mice (E2FAD, *n* = 4; E3FAD, *n* = 5; E4FAD, *n* = 5). Initially, we focused on assessing the region-specific Aβ deposition in these sections by measuring Aβ (MOAB2) immunoreactivity. We quantified the following parameters from selected individual Aβ-positive deposits in the subiculum and deep layers of the cortex: plaque area, fluorescence intensity and plaque type.

**Figure 1:**
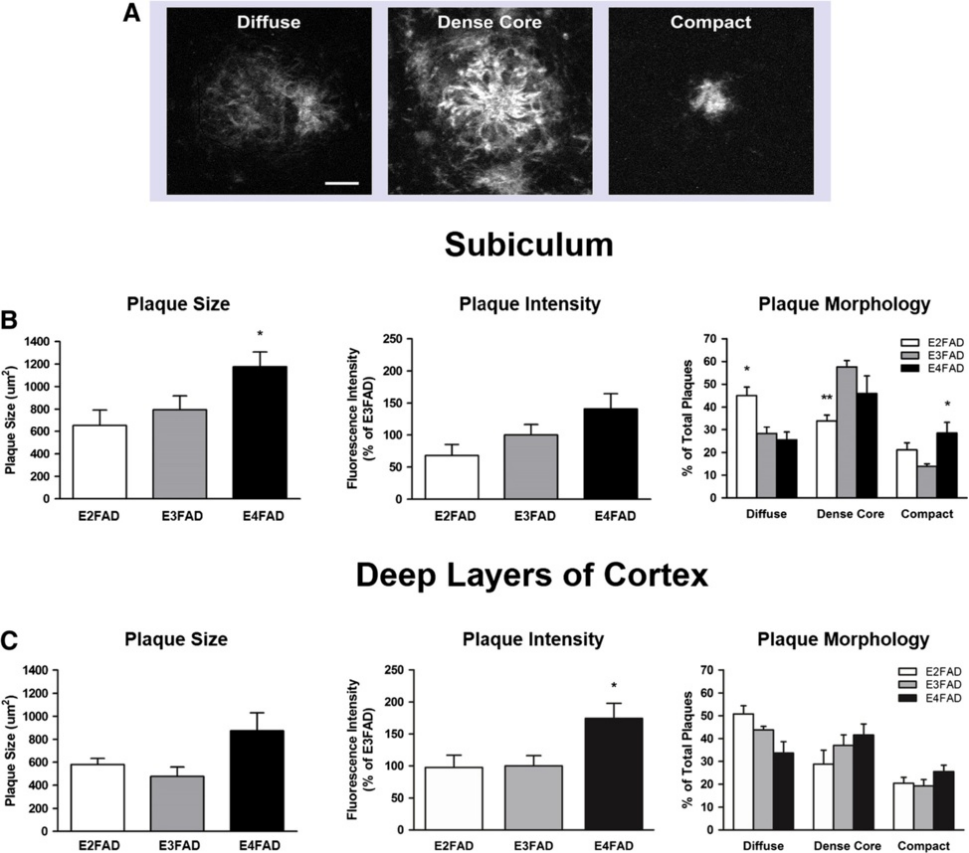
**APOE genotype affects Aβ plaque morphology in EFAD subiculum and deep layers of cortex.** Morphologically distinct Aβ plaques were visualized in the subiculum and deep layers of the cortex of 6-month-old EFAD mice using immunofluorescence staining with Aβ antibody MOAB2. **(A)** Heterogeneous plaque types were evident in EFAD brains and grouped into three categories: diffuse, dense core and compact. Scale bar: 12 μm. **(B)** Subiculum. Left: E4FAD mice exhibited larger plaques on average than E3FAD mice. Center: Fluorescence intensity of plaques did not differ by APOE genotype. Right: E2FAD mice exhibited a greater percentage of diffuse plaques than E3FAD or E4FAD mice, and a lower percentage of dense core plaques than E3FAD mice. Interestingly, E4FAD mice exhibited an increased percentage of compact plaques in the subiculum. **(C)** Deep layers of the cortex. Left: Plaque size did not differ by APOE genotype. Center: Fluorescence intensity of deep cortical plaques was highest in E4FAD mice. Right: E2FAD mice had a greater percentage of diffuse plaques in this region than E4FAD mice. No differences were detected between APOE genotype for dense core or compact plaques. One-way ANOVA, * *P* < 0.05. Two-way ANOVA, * *P* < 0.05, ** *P* < 0.01.

In the subiculum, we found a significant effect of APOE genotype on plaque size (*F*_(2,11)_ = 4.30, *P* < 0.05), with *post hoc* comparisons revealing significantly larger plaques sampled in E4FAD sections compared to E2FAD sections (*P* < 0.05) (Figure 1B, left). MOAB2 fluorescence intensity was not affected by APOE genotype (*F*_(2,11)_ = 3.25, *P* = 0.078) in this brain region (Figure 1B, center). Finally, we defined Aβ deposits as diffuse, dense core or compact plaques, expressed the total number of diffuse, dense core and compact plaques as a percentage of total plaques, and compared them within and between APOE genotypes (Figure 1A and 1B right). Two-way ANOVA revealed a significant main effect of plaque type (*F*_(2,33)_ = 26.65, *P* < 0.001) on the distribution of plaques in all EFAD groups, with a greater number of dense core plaques (45.83 ± 0.3%) in the subiculum than diffuse plaques (32.9 ± 0.5%) or compact plaques (21.10 ± 0.5%). There was a higher percentage of diffuse plaques in E2FAD sections compared to E3FAD sections (*P* < 0.05) (E2FAD vs E3FAD; 45.0 ± 3.8% vs 28.3 ± 2.8%) and E4FAD sections (25.5 ± 3.5%) (*P* < 0.01) (Figure 1B, right). There were also significantly fewer dense core plaques in E2FAD sections (33.9 ± 2.6%) than E3FAD sections (57.6 ± 2.8%) (*P* < 0.01), and a higher percentage of compact plaques in E4FAD sections (28.5 ± 4.8%) than E3FAD sections (13.9 ± 1.1%) (*P* < 0.05). Thus, in the subiculum, APOE2 was associated with more diffuse plaques, and APOE4 with more compact plaques, compared to APOE3.

Within the deep layers of the cortex, we did not detect EFAD group differences in plaque size (*F*_(2,11)_ = 3.45, *P* = 0.068) (Figure 1C, left). However, APOE genotype did affect MOAB2 fluorescence intensity (*F*_(2,11)_ = 4.88, *P* < 0.05), with E4FAD plaques exhibiting higher MOAB2 positive staining compared to E3FAD plaques (*P* < 0.05) (Figure 1C, center). A two-way ANOVA to assess the distribution of plaque types in the cortex revealed a significant main effect of plaque type (*F*_(2,33)_ = 21.43, *P* < 0.001) for each EFAD group, with higher percentages of diffuse plaques (42.7 ± 5.0%) and dense core plaques (35.8 ± 3.7%) in EFAD sections than compact plaques (21.7 ± 1.9%) (Figure 1C, right). Interestingly, a higher percentage of diffuse plaques was again seen in E2FAD sections (50.8 ± 3.6%) compared to E4FAD sections (33.6 ± 5.0%).

These data identify two sites of robust extracellular Aβ deposition in the brains of 6-month-old EFAD mice. APOE genotype had an effect on plaque characteristics in both the subiculum and deep layers of the cortex, which is consistent with previous findings for these mice [32].

### Glial activation is increased in the EFAD subiculum and cortex, while E4FAD mice exhibit elevated IL-1β levels

**Figure 2:**
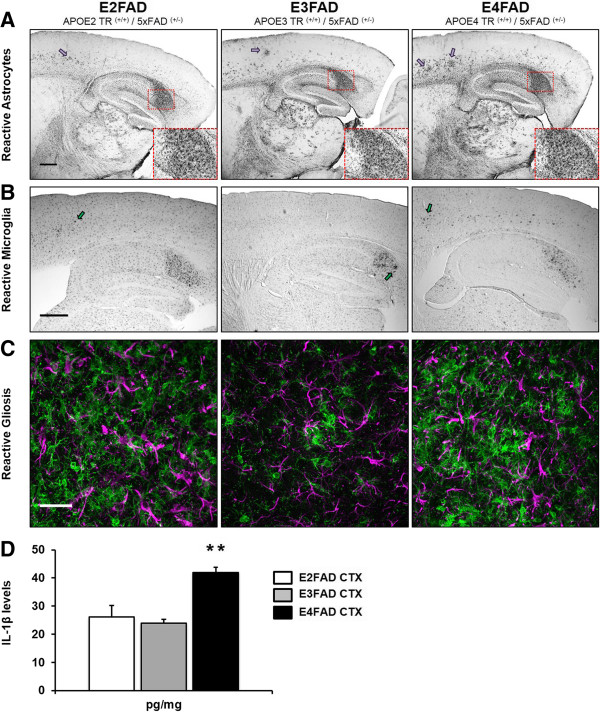
**Glial activation is increased in the EFAD subiculum and cortex, while E4FAD mice exhibit elevated IL-1β levels.** Astrocytes and microglia were visualized in sagittal brain sections of 6-month-old EFAD mice using immunohistochemistry. Initial DAB staining revealed prominent gliosis in two regions of the brain: the subiculum and deep layers of the cortex. **(A)** Extensive GFAP-immunoperoxidase staining was evident in the subiculum of all EFAD mice. In addition, dystrophic astrocytes could be seen throughout the deep layers, but not in superficial layers, of the EFAD cortex. Insets: 20× magnification of the subiculum (dashed red box). Magenta arrows: Clusters of dystrophic astrocytes in the cortex. Scale bar: 500 μm. **(B)** Activated microglial cells were clearly visible in the subiculum of all EFAD mice and were present in deep cortical layers as well. E4FAD sections exhibited more activated microglia in the deep cortex than E2FAD and E3FAD sections. Green arrows: Activated microglia. Scale bar: 500 μm. **(C)** Double immunofluorescence staining for GFAP (magenta) and Iba1 (green) confirmed region-specific glial activation in EFAD brains. Representative images of EFAD subicula are shown. Scale bar: 30 μm. **(D)** Levels of the pro-inflammatory cytokine IL-1β were measured using ELISA in 6-month-old EFAD cortex (CTX) extracts. E4FAD sections exhibited significantly higher levels of IL-1β than E3FAD sections. One-way ANOVA, ** *P* < 0.01.

Aβ accumulation and deposition in the brain can lead to reactive gliosis [39]. This term refers to the physiological activation of glial cells in response to focal tissue damage, and is characterized by specific structural and functional changes to glial cells that mediate the neuroinflammatory response. Astrocytes and microglia are two important glial cell types involved in Aβ-associated glial activation [40]. Therefore, we assessed glial activation in 6-month-old EFAD mice by analyzing the distribution and numbers of dystrophic astrocytes and microglial cells in the brain.DAB-immunoperoxidase staining for GFAP (astrocytes) was performed on sagittal brain sections from E2FAD, E3FAD and E4FAD mice. Intense GFAP staining was evident in the subiculum of all EFAD mice (Figure 2A), with numerous reactive astrocytes having dystrophic processes in the region (Figure 2A, inset). Sparse, but intense, GFAP staining was also evident in deep layers of the cortex, where clusters of reactive astrocytes were detected (Figure 2A, magenta arrows). Double immunofluorescence staining for GFAP and MOAB2 confirmed that plaques were at the center of reactive astrocytic clusters in deep cortical layers (data not shown). DAB staining for Iba1 revealed a similar pattern for microglia in the EFAD subiculum, although reactive microglia appeared more frequent in the E4FAD and E2FAD cortices than E3FAD cortices (Figure 2B, green arrows). Double immunofluorescence staining for reactive astrocytes and microglia was performed on adjacent EFAD sections to visualize reactive gliosis in these regions. Dystrophic astrocytes and microglial reactivity were clearly evident in the subiculum, with both glial cell types exhibiting strong intensity and activated morphologies (Figure 2C).

IL-1β is a member of the interleukin-1 family of cytokines and is an important microglia-derived mediator of neuroinflammation. Six-month-old 5xFAD mice show significant activation of IL-1β in the brain, suggestive of an Aβ-dependent neuroinflammatory response [41]. APOE genotype exerts an influence over inflammatory responses in the brain independent of AD pathology [42]. APOE4 is associated with upregulated gene expression of pro-inflammatory cytokines following traumatic brain injury and lipopolysaccharide (LPS) treatment compared to APOE3 [30,43]. To test whether APOE genotype affects functional aspects of glial activation in EFAD mice, we measured levels of the pro-inflammatory cytokine IL-1β in TBS-soluble fractions of 6-month-old EFAD cortices (E2FAD, *n* = 4; E3FAD, *n* = 4; E4FAD, *n* = 4) (Figure 2D). One-way ANOVA revealed a significant effect of APOE genotype (*F*_(2,9)_ = 12.66, *P* < 0.01) on IL-1β levels. A significant elevation in IL-1β was detected in the E4FAD cortex (41.9 ± 1.9 pg/mg), representing an increase of nearly twofold compared to E3FAD samples (23. 8 ± 1.5 pg/mg) and E2FAD samples (26.0 ± 4.1 pg/mg).

Thus, morphological characteristics of reactive gliosis were revealed in the adult EFAD subiculum and deep layers of the cortex. Initial DAB-immunoperoxidase staining showed strong glial immunoreactivity in the subiculum of all mice. Increased clusters of astrocytes and an increased number of reactive microglial cells were found in deep layers of the E4FAD cortex compared to the E3FAD cortex. Z-series projections of double-stained images revealed highly dystrophic astrocytic and microglial processes. In addition, we found elevated levels of pro-inflammatory IL-1β in the E4FAD cortex.

### E4FAD mice exhibit increased Aβ-associated microglial reactivity in deep layers of the cortex

**Figure 3:**
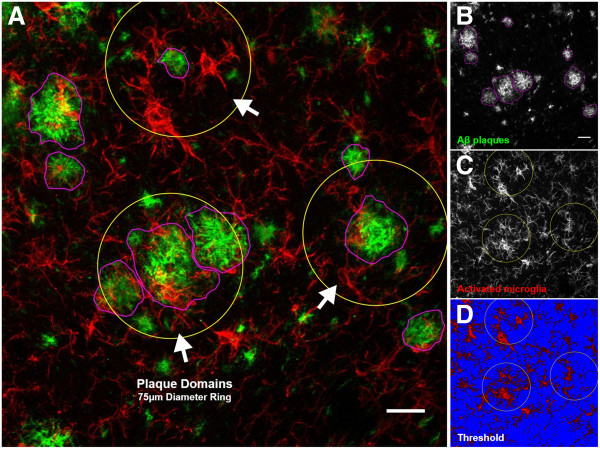
**Confocal microscopy analysis of Aβ-associated microglial activation in EFAD mice.** Sagittal brain sections from 6-month-old EFAD mice were analyzed for Aβ deposition and associated microglial activation in two regions of interest, the subiculum and deep layers of the cortex. Reactive microglial cells within the vicinity of Aβ plaques consistently exhibited an amoeboid-like shape with dystrophic processes. **(A)** Two-dimensional projection image (approximately 20 μm, *z*-axis) of a E3FAD subiculum double stained for Aβ_1–42_ (MOAB2) and activated microglia (Iba1). Image overlays depict metrics used to characterize and quantify Aβ deposition and associated microglial activation. White arrows*:* Examples of plaque domains. **(B)** Grayscale image of Aβ plaques chosen for analysis. Plaque types, size of plaques and MOAB2 fluorescence intensity were quantified. Magenta: Traced plaques. **(C)** Grayscale image of activated microglia with plaque domain overlays. The number of microglia cell bodies within plaque domains were collected and quantified along with Iba1 fluorescence intensity. Yellow ring: Plaque domains (4.42 mm^2^). **(D)** Thresholding was performed within plaque domains to quantify the percentage area occupied by microglia cell bodies and processes. Representative minimum threshold value of 35 (0 to 255 brightness scale) depicted. Blue: Background. Scale bars: 20 μm.

Our region-specific glial staining and ELISA data revealed increased microglial reactivity in the E4FAD mouse brain. To determine whether APOE genotype had an effect on Aβ-associated microglial reactivity independent of plaque load [32], we analyzed microglial activation within the vicinity of individual Aβ plaques. Using the MOAB2/Iba1 double-stained tissue sections (Figures 1 and 3A,B,C), we established domains around select plaques having little to no overlap with other domains (Figure 3A,C). We then isolated the Iba1-positive fluorescence images from the original double-stained sets, superimposed plaque domains on these single-channel images, and quantified the following parameters within the plaque domains: average number of microglial cells, Iba1 fluorescence intensity and percentage area occupied by the microglia (Figure 3C,D).

**Figure 4:**
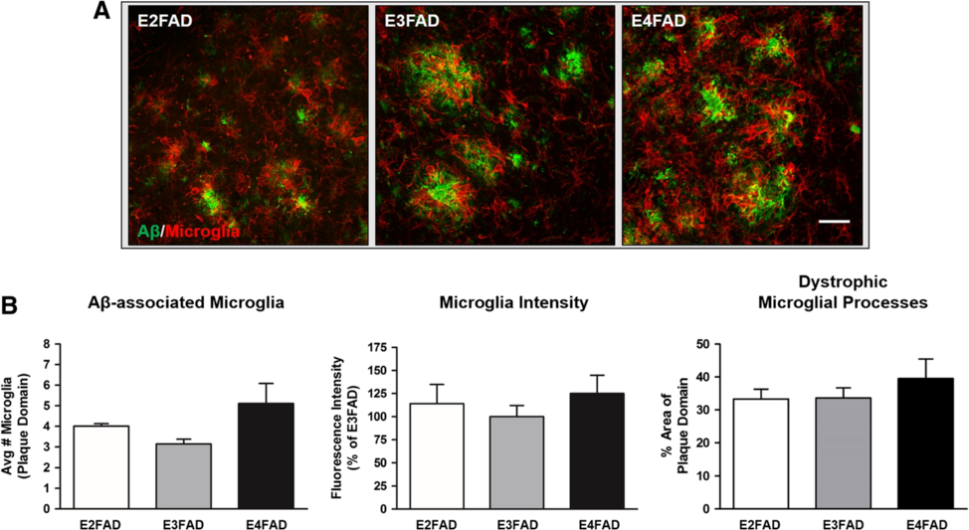
**Aβ deposition in the subiculum is associated with high levels of microglial reactivity.** Double immunofluorescence staining of 6-month-old EFAD subicula revealed significant Aβ deposition and elevated microglial activation in each group. **(A)** Representative images of fluorescence staining in EFAD subiculum. Aβ-associated microglial activation was assessed for each APOE genotype (*n* = 4 or 5 mice/APOE genotype). Scale bar: 30 μm. **(B)** Microglial density, fluorescence intensity and percentage area of the plaque domain did not differ between APOE genotypes. Within the subiculum, the reactive microglia in each group occupies a large percentage of the plaque domains relative to the deep cortex.

Representative images of Aβ-associated microglial reactivity are shown for E2FAD, E3FAD, and E4FAD subiculum (Figure 4A). Within this region of the EFAD brain, we did not detect differences in the average number of microglial cells surrounding plaques (*F*_(2,11)_ = 2.64, *P* > 0.05), Iba1-positive fluorescence intensity (*F*_(2,11)_ = 0.54, *P* > 0.05) or percentage area of plaque domain occupied by microglia (*F*_(2,11)_ = 0.64, *P* > 0.05) (Figure 4B).

**Figure 5:**
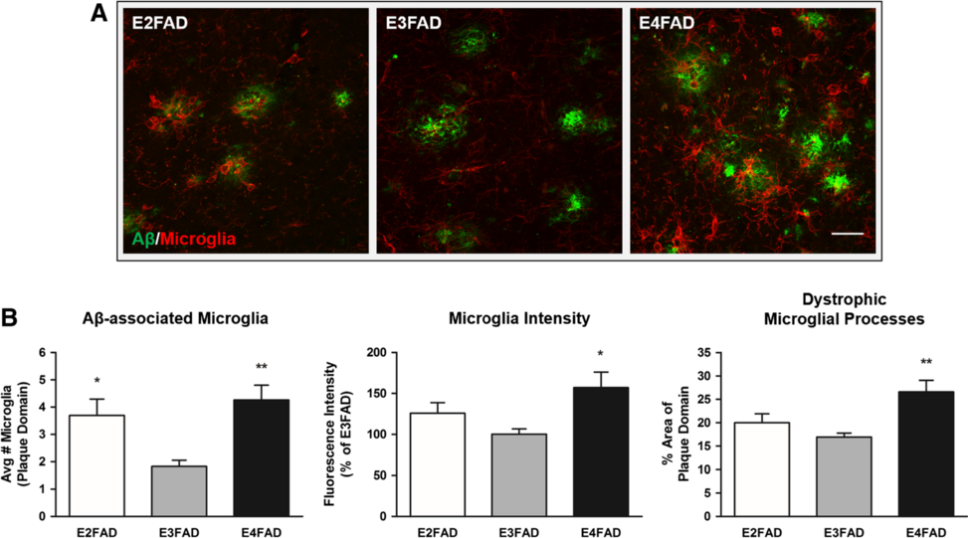
**APOE4 is associated with increased microglial reactivity to Aβ in deep layers of the cortex.** Morphological profiles of reactive microglia within deep cortical Aβ plaque domains were elevated in E4FAD mice. **(A)** Representative double-stained images of Aβ plaques and microglial activation in deep layers of EFAD cortices (*n* = 4 or 5 mice/APOE genotype). Scale bar: 30 μm. **(B)** Left: E4FAD and E2FAD mice exhibited increased reactive microglial density within Aβ plaque domains. Center: Fluorescence intensity of the microglia was elevated in E4FAD mice relative to E3FAD mice. Right: E4FAD microglia occupied a larger percentage of plaque domains than E3FAD microglia. One-way ANOVA, * *P* < 0.05, ** *P* < 0.01.

However, in the deep layers of the cortex, activated microglial profiles were elevated in E4FAD sections compared to E3FAD sections (Figure 5A). We found a significant effect of APOE genotype on the average number of microglial cells surrounding plaques (*F*_(2,11)_ = 7.90, *P* < 0.01), with E4FAD mice exhibiting a 2.3-fold increase compared to E3FAD mice (*P* < 0.01) (Figure 5B, left). Interestingly, Aβ-associated microglial cell counts were also elevated within E2FAD plaque domains compared to E3FAD plaque domains (*P* < 0.05). In addition, differences in Aβ-associated microglia intensity (*F*_(2,11)_ = 4.36, *P* < 0.05) were detected between EFAD groups, with increased staining intensity present in E4FAD plaque domains relative to E3FAD plaque domains (*P* < 0.05) (Figure 5B, center). When measuring the percentage area of plaque domains occupied by microglia, we found a significant effect of APOE genotype (*F*_(2,11)_ = 7.35, *P* < 0.01) (Figure 5B, right). *Post hoc* tests revealed a higher percentage of dystrophic microglia occupying E4FAD plaque domains than E3FAD plaque domains (*P* < 0.01). The E2FAD and E3FAD sections analyzed had similar percentages of total microglial staining within plaque domains (E2FAD, 20.0 ± 1.2% vs E3FAD, 16.9 ± 0.8%), despite E2FAD plaque domains containing more microglial cells than E3FAD plaque domains (Figure 5B, left).

With this detailed analysis of EFAD brains, we found that Aβ-associated microglia reactivity was not affected by APOE genotype in the subiculum. However, we found that APOE genotype affected morphological characteristics of microglial reactivity, with several measures of increased microglial activity detected within E4FAD cortical plaque domains, independent of overall plaque load.

## Discussion

APOE-dependent differences in the innate inflammatory response to Aβ may partially explain the differential risk for AD caused by APOE genotype [22]. In the present study, we investigated whether APOE alleles affect glial markers of Aβ-associated neuroinflammation in a mouse model of Aβ deposition. First, we found that E4FAD mice exhibited larger, more intensely stained Aβ plaques in the brain compared to E2FAD and E3FAD mice. E4FAD mice also had a greater number of compact plaques in the subiculum than E3FAD mice. Initial immunostaining experiments revealed activated astrocytes and microglial cells in the subiculum and deep layers of the cortex, and an ELISA revealed significantly elevated levels of IL-1β in the E4FAD cortex. These findings prompted our investigation into microglial reactivity surrounding plaques in the subiculum and deep cortex. We did not detect APOE-dependent differences in microglial reactivity within the subiculum, perhaps related to the very intense deposition of Aβ in the region for all groups. In the deep layers of the cortex, however, APOE genotype had a significant effect on Aβ-associated microglial activation. E4FAD mice exhibited higher numbers of reactive microglial cells surrounding Aβ plaques and increased signs of activation compared to E3FAD mice. These data support the idea that Aβ-associated microglial activation, an important component of the neuroinflammatory response in AD, is augmented by the APOE4 genotype.

5xFAD mice exhibit rapid amyloid deposition and pathology in the brain. Extracellular amyloid can be detected as early as 2 to 4 months of age in the subiculum and deep layers of the cortex [32,33]. By introducing human APOE alleles into the 5xFAD model, the effects of APOE on amyloid-associated pathological changes in the adult brain can be investigated. The EFAD mouse model is particularly good for examining the effects of APOE genotype on Aβ-associated inflammation. In our experiments, we were first interested in characterizing extracellular Aβ plaques in the brains of adult EFAD mice. We found mostly large Aβ plaques in the E4FAD subiculum, with no group differences in MOAB2 staining intensity (Figure 1B). Our morphological results support findings showing a higher percentage of compact plaques in the subiculum of 6-month-old E4FAD mice compared to E3FAD mice (Figure 1B) [32]. We also found a higher percentage of diffuse plaques in the subiculum of E2FAD mice compared to E3FAD mice, though no differences were found between these two groups previously [32]. This is likely due to the antibody used to stain plaque deposits. Here we used MOAB2, a pan-specific Aβ antibody that recognizes several conformational species of Aβ_1–42_[36], while the previous study [32] used thioflavin-S to stain beta-sheet-rich amyloid fibrils in plaques. MOAB2-stained Aβ plaques were clear and could easily be distinguished from one another using our methods (Figure 1A). Importantly, the percentage of diffuse plaques in the E2FAD subiculum was greater than in the E4FAD subiculum, which supports previous plaque morphology data for these mice [32]. In deep layers of the cortex, APOE genotype did not influence plaque size, though we did detect increased MOAB2-staining intensity in E4FAD plaques compared to E3FAD plaques (Figure 1C). The significance of greater plaque fluorescence intensity on glial activation is unclear, but may be related to changes in microglial reactivity surrounding plaques that we observed in our studies. More research on Aβ deposition and plaque-associated neuroinflammation in the EFAD model is needed, with an emphasis on microglial-secreted pro-inflammatory and anti-inflammatory signaling molecules that affect nearby cells.

Microglial cells are the resident macrophages in the CNS and are responsible for several functions of the innate immune response [44,45]. Notably, the microglia survey the brain parenchyma for foreign substances and cellular debris, shifting their activation states to phagocytose the material and clear the microenvironment. In our experiments, both DAB-immunoperoxidase and double immunofluorescence staining revealed aggressive astrocytosis and microgliosis in the subiculum of E2FAD, E3FAD and E4FAD mice (Figure 2A,C), while reactive microglia were most visible in the cortex of E4FAD and E2FAD mice (Figure 2B). Age-matched cortical samples were analyzed for total IL-1β levels by ELISA. We detected increased levels of pro-inflammatory IL-1β in the E4FAD cortex compared to the E3FAD cortex (Figure 2D). These data led us to investigate morphological markers of microglial reactivity proximal to Aβ plaques in both brain regions. Reactive microglia in AD brains have been found to be localized to Aβ plaques [6,46], and *in vitro* studies have shown that Aβ directly activates the microglia to produce IL-1β, reactive oxygen species and tumor necrosis factor α [47-49]. Interestingly, APOE is associated with Aβ-independent immunomodulatory functions *in vitro*. ApoE suppresses the LPS-stimulated release of tumor necrosis factor α in primary glial cultures, and attenuates microglial activation by secreting derivatives of amyloid precursor protein in an isoform-dependent manner [50,51]. The anti-inflammatory effects of APOE may be mediated through low-density lipoprotein receptor-related protein (LRP1) on microglia and subsequent suppression of the c-Jun N-terminal kinase pathway [52,53]. In animal models, apoE deficiency is associated with poor recovery from CNS injuries involving neuroinflammation, demonstrating its important immunomodulatory role in the brain [54,55]. Our experiments using the EFAD model of Aβ deposition have provided additional insights on the role APOE plays in the neuroinflammatory response to AD pathological brain changes.Analyzing morphological markers of microglial activation within defined Aβ domains allowed us to control for the effects of APOE on amyloid accumulation. Since APOE4 is associated with higher levels of amyloid deposition in both humans and mouse models, higher levels of global inflammation would be expected for mice with APOE4. In our analysis, we measured microglial reactivity within the microenvironment of randomly selected Aβ plaques (Figure 3). To fit our selection criteria, plaques needed to be clearly visible, larger than 10 μm in diameter and easily discerned from other plaque types. Every effort was made to select individual plaques for analysis that were at least 35 μm from another plaque, which we determined was the maximum distance for which we could accurately judge plaque-specific microglia reactivity. This analysis proved challenging in the subiculum, where each EFAD group exhibited aggressive Aβ deposition, and thus plaques were more likely to be near one another. Our results describing Aβ-associated microglia reactivity in the subiculum may reflect this, as we did not detect APOE genotype differences in Aβ-associated microglia reactivity (Figure 4). We hypothesize that measuring Aβ-associated microglial reactivity in the subiculum of younger EFAD mice would remove the effects of proximal Aβ plaques, as Aβ deposition would be reduced in the region and more accurate assessments of glial activation could be made. Nonetheless, we were able to measure markers of microglia reactivity readily within the microenvironment of plaques found in the deep layers of the cortex (Figure 5). We detected an increased density of reactive intensely stained microglia surrounding individual plaques in E4FAD sections compared to E3FAD sections. Interestingly, E2FAD mice also exhibited increased numbers of microglial cells surrounding plaques, though these cells did not appear as activated as those from E4FAD mice. This conclusion is reflected in the low Iba1 fluorescence intensity and a smaller percentage area of the plaque domain occupied by cell soma and processes. Thus, the higher domain area occupied by the microglia in E4FAD mice likely reflects swollen cell somas and hypertrophic processes, and not simply an increase in microglia density (Figure 5B). Collectively, these data suggest that APOE genotype differentially affects activation of microglia in response to Aβ deposits, and that morphological profiles of microglial reactivity can serve as a useful measure of Aβ-driven neuroinflammation.

The effects of APOE genotype on the increased microglial activation in E4FAD mice may be due to differences in the form of apoE or in the amount of apoE. Several studies have reported lower levels of total apoE in the brains of APOE4 knock-in mice [56-58]. We previously measured brain apoE levels in EFAD mice using a three-step sequential protein extraction protocol (using TBS, TBS-Triton X-100 and formic acid) [32]. Levels of apoE4 were selectively lower than apoE3 in the TBS-Triton X-100 fraction, suggesting that the lower levels of apoE4 may be due to the fact that the apoE is less lipidated. Lower levels of properly lipidated apoE could lead to less receptor binding and attenuated inhibition of microglial activation at Aβ plaques [52,53]. We previously found that APOE knock-out and APOE4 knock-in mice have higher levels of LPS-induced neuroinflammation and impaired synaptic viability than APOE3 mice, suggesting that the loss of apoE contributes to increased neuroinflammation [30]. These data are consistent with *in vitro* and *in vivo* data showing that Aβ-independent neuroinflammation is higher with APOE4 than APOE3 [26,58,59]. For a review, see [60].

The type and amount of apoE also can affect glial responses to other forms of Aβ, in addition to extracellular deposits. Our previous data demonstrate that levels of soluble Aβ42 and soluble oAβ are higher in both the hippocampus and cortex of E4FAD mice compared to E3FAD mice [32]. Analysis of EFAD mice suggested that reduced lipidation of apoE4 results in lower levels of the apoE4/Aβ complex, resulting in increased levels of soluble oAβ [31,61]. However, dissecting the effect of APOE-genotype-modulated extracellular Aβ vs soluble oAβ on microglial activation *in vivo* is complex, as soluble Aβ and insoluble Aβ exist in a dynamic compartmentalization [62], and oAβ levels are higher in microenvironments surrounding amyloid plaques [63]. Thus, both forms of Aβ likely contribute to microglial activation. Collectively, these data support the hypothesis that lipid-poor apoE4 may result in a loss of function not only on Aβ clearance, but also on Aβ-independent and Aβ-driven neuroinflammation, both of which are a focus of our ongoing studies.

In summary, we have shown that APOE genotype differentially impacted Aβ-plaque-associated microglial activation in the brains of EFAD mice. APOE4 increased levels of IL-1β and negatively affected morphological profiles of microglial reactivity within cortical Aβ plaque domains. In addition, APOE genotype affected characteristics of Aβ plaques in the EFAD subiculum, supporting previously reported data describing Aβ deposition in this model. Our data support the use of the EFAD transgenic mouse model for studies of Aβ-associated neuroinflammation, and demonstrate the need for APOE-targeted therapeutics in AD aimed at regulating neuroinflammation.

## Abbreviations

Aβ: amyloid beta; AD: Alzheimer’s disease; ANOVA: analysis of variance; APOE: apolipoprotein E; APP: amyloid precursor protein; BSA: bovine serum albumin; CNS: central nervous system; ELISA: enzyme-linked immunosorbent assay; IL: interleukin; LPS: lipopolysaccharide; oAβ: oligomeric amyloid beta; PBS: phosphate-buffered saline; PS1: presenilin-1; TBS: tris-buffered saline; TBST: Triton X-100 in tris-buffered saline.

## Competing interests

The authors declare that they have no competing interests.

## Authors’ contributions

GAR participated in the design of the study, performed immunohistochemical experiments, image analyses, and drafted the manuscript. LMT performed EFAD brain harvesting, biochemical analyses, and assisted in preparation of the manuscript. MJL and GWR conceived of the study, participated in its design, and helped draft the manuscript. All authors read and approved the final manuscript.
